# Awareness and perceptions of artificial intelligence among pulmonologists and thoracic surgeons: a national survey

**DOI:** 10.1186/s13019-026-04672-y

**Published:** 2026-08-25

**Authors:** Gizem Özçıbık Işık, Neslihan Özçelik, Hasan Volkan Kara

**Affiliations:** 1 Department of Thoracic Surgery, Yedikule Chest Diseases and Thoracic Surgery Education and Research Hospital, İstanbul, Türkiye; 2 Department of Pulmonary Medicine, Recep Tayyip Erdogan University Medical Faculty, Rize, Türkiye; 3 Department of Thoracic Surgery, Istanbul University Cerrahpasa-Cerrahpasa Medical Faculty, İstanbul, Türkiye

**Keywords:** Artificial Intelligence, Pulmonologists, Thoracic Surgeons, Survey

## Abstract

**Background:**

Artificial intelligence (AI), a set of algorithms that mimic human intelligence, is increasingly being used in the healthcare field.

**Objective:**

This study aims to evaluate the awareness, expectations, and concerns of pulmonologists and thoracic surgeons regarding AI, as well as to assess their level of adaptation to these technologies.

**Methods:**

A 23-item online survey was administered to specialists and residents in the fields of pulmonology and thoracic surgery between January and May 2025. Participation was voluntary, and informed consent was obtained from all respondents. Data were analyzed using SPSS version 27.0, with a p-value of < 0.05 considered statistically significant.

**Results:**

Among the 302 participants, 65.6% were pulmonologists and 34.4% were thoracic surgeons. A total of 85.4% believed that AI will be actively used in clinical practice, and 67.5% believed that AI would reduce the need for physician workforce. However, 59.3% considered AI less successful than physicians in diagnosis, and 65.6% in treatment. In addition, 86.8% emphasized the need for legal regulations, and 58.3% expressed concerns about data privacy.

**Conclusions:**

Participants generally expressed a positive attitude toward AI, although they remained cautious due to ethical, legal, and technical concerns. Comprehensive regulations regarding education, legislation, and data security are needed to ensure the safe and effective integration of AI into clinical practice.

**Supplementary Information:**

The online version contains supplementary material available at https://doi.org/10.1186/s13019-026-04672-y.

## Introduction

Artificial intelligence (AI) is a set of algorithms designed by imitating human learning and decision processes and neural structures, to create intelligent machines [1–3]. The primary aim for using AI applications is to simplify and enhance the efficiency of daily life in multiple ways [1]. Artificial intelligence officially appeared in the scientific and public domain with the Turing Test in the 1950s [2, 4, 5]. Today, AI is utilized in numerous fields, and various language models are becoming part of everyday life, providing diverse conveniences [6, 7]. These applications imitate human cognitive functions such as language comprehension, visual perception, and decision-making. Their foundations were laid by the ELIZA chatbot in the 1960s [2, 6, 7]. AI models can be developed using various algorithms, including machine learning and deep learning techniques [8, 9].

AI applications are increasingly being used in healthcare services [10]. The most common uses of AI in medicine include digitizing electronic health record data, identifying data trends, improving time management, enhancing statistical modeling, supporting decision-making and risk estimation, solving complex problems, and facilitating thus evaluating medical training [2, 10, 11]. In clinical medicine, AI is most frequently applied in standardized image pictures using disciplines such as radiology, pathology, and dermatology [12–14]. Among AI-based diagnostic and interventional radiology applications were developed using image processing techniques, the most commonly used for neuro, thoracic, breast, and musculoskeletal radiology [15, 16]. Radiologic modalities employed in these applications include computed tomography (CT), magnetic resonance imaging (MRI), and X-ray imaging [15]. Analyses can be conducted on images obtained from these modalities based on their radiomics properties [15, 16]. Radiomics refers to the extraction and analysis of quantitative data from existing radiological images—features that are not discernible to the naked eye—to obtain diagnostic, prognostic, or predictive information [16]. Although radiomics was initially used in oncology as the start, its success has also been demonstrated in cases of pulmonary fibrosis, COVID-19 pneumonia, and neurodegenerative diseases [16].

The use of AI models in thoracic surgery and pulmonary medicine is also increasing [17, 18]. Image processing techniques are utilized for lung parenchymal nodule monitoring and cancer risk assessment, also COVID-19 pneumonia diagnosis, as a decision-support mechanism for clinicians [16, 19]. Recent studies have shown that endobronchial and transbronchial cytologic samples can be successfully evaluated using AI to determine sample adequacy and establish a histopathological diagnosis [19]. The effectiveness of AI-based applications has also been demonstrated in the interpretation of pulmonary function tests and the evaluation of polysomnography [19]. Furthermore, AI-based models and scoring systems have been developed for risk and prognosis prediction in thoracic surgery, and AI has made significant contributions to the advancement of robotic surgery [20].

Current literature indicates that physicians generally perceive the use of AI in medicine as beneficial [21]. However, due to the high rate of inaccuracy associated with incomplete data, the superiority of AI remains a subject of debate among physicians [21]. In certain fields, some physicians believe that their tasks may eventually be fully performed by AI, potentially leading to professional displacement [21]. Overall, medical students share the view that AI is beneficial for medical education and should be integrated into training programs [22]. Physicians express a range of advantages and disadvantages regarding AI applications and their effect in medical life [21–22].

In this study, we aimed to assess the awareness and level of self-perceived knowledge of thoracic surgeons and pulmonologists to AI. We tried to identify their expectations and concerns, and to evaluate their adaptability to technological advancements.

## Materials and methods

The questionnaire was developed by the investigators based on a review of previously published studies evaluating physicians’ knowledge, attitudes, and awareness regarding the use of artificial intelligence in healthcare and was adapted to the objectives of the present study. The draft questionnaire was reviewed by experienced pulmonologists and thoracic surgeons to assess its content validity, scope, and clarity, and appropriate revisions were made based on their recommendations. Subsequently, the questionnaire was pilot-tested on five physicians to evaluate its comprehensibility and feasibility before finalization. Internal consistency was assessed using Cronbach’s alpha coefficient, which was 0.802, indicating good internal consistency. The survey questions were non-directive and aimed to assess general attitudes (Table 1 and Supplementary Table 1).

**Table 1:** Survey questions and participants’ responses

Questions	*n* (%)
**Q 1: Your age?**	
A) < 35 B) 36–40 C) 41–45 D) 46–50 E) 51–55 F) 56–60 G) 61–65 H) > 65	**129 (42.7%)** 43 (14.7%) 42 (13.9%) 37 (12.3%) 28 (9.3%) 16 (5.3%) 6 (2%) 1 (0.3%)
**Q 2: Your gender?**	
A) Female B) Male	153 (50.7%) 149 (49.3%)
**Q 3: Your department?**	
A) Thoracic Surgery B) Pulmonary Medicine	104 (34.4%) 198 (65.6%)
**Q 4: Institution you work for?**	
A) State Hospital B) Education and Research Hospital C) University Hospital D) Private Institutions and Organizations	39 (12.9%) **118 (39.1%)** **113 (37.4%)** 32 (10.6%)
**Q 5: How many years have you been working in your field?**	
A) < 5 Years B) 5–10 Years C) 10–15 Years D) 15–20 Years E) > 20 Years	**108 (35.8%)** 61 (20.2%) 49 (16.2%) 25 (8.3%) 59 (19.5%)
**Q 6: Your title?**	
A) Medical Specialization Student B) Specialist Physician C) Assistant Lecturer D) Associate Professor E) Professor	**98 (32.5%)** **94 (31.1%)** 36 (11.9%) 32 (10.6%) 42 (13.9%)
**Q 7: How would you describe your knowledge about artificial intelligence? (0–5)**	
A) 0- No idea B) 1- Little C) 2- Average D) 3- Above Average E) 4- Good F) 5- Excellent	11 (3.6%) 81 (26.8%) **118 (39.1%)** 50 (16.6%) 32 (10.6%) 10 (3.3%)
**Q 8: Have you ever used AI applications in your clinical practice?**	
A) Yes B) No	**125 (41.4%)** 177 (58.6%)

The survey was conducted between January and May 2025. Participation in the online survey was voluntary, and participants were informed about the content and purpose of the study in advance. Informed consent was obtained prior to participation, and all responses were collected anonymously. The survey was created using the Google Forms platform and distributed to pulmonology and thoracic surgery residents and specialists through professional WhatsApp groups and e-mail. Participation was entirely voluntary. A convenience sampling strategy was used. Because the survey link was disseminated through multiple electronic communication channels, the total number of physicians invited to participate could not be determined, and therefore the response rate could not be calculated.

Statistical analyses were performed using SPSS version 27.0 (IBM Corp., Armonk, NY, USA). Descriptive statistics were presented as numbers, percentages, means, and standard deviations. Subgroup analyses were planned according to age group, academic title, and specialty. Categorical variables were compared using the chi-square test. Odds ratios (ORs) with 95% confidence intervals (CIs) were calculated for binary comparisons. Reference groups for OR calculations were defined before the analyses and are clearly indicated in the Results section. To identify factors independently associated with a positive overall attitude toward artificial intelligence, a multivariable binary logistic regression analysis was performed. Statistical significance was defined as *p* < 0.05.

## Results

A total of 302 physicians participated in the study. Among them, 153 were female (50.7%) and 149 were male (49.3%). Regarding age distribution, 42.7% of participants were under 35 years of age, 28.1% were between 36 and 45, 21.6% were between 46 and 55, and 7.6% were over 56 years old. Of the participants, 65.6% worked in the field of pulmonology, and 34.4% in thoracic surgery. The distribution by type of institution was as follows: 39.1% were employed in training and research hospitals, 37.4% in university hospitals, 12.9% in public hospitals, and 10.6% in private institutions and organizations.

Based on length of professional experience, 35.8% of participants had been working in their field for less than five years, 20.2% for 5–10 years, 16.2% for 10–15 years, 8.3% for 15–20 years, and 19.5% for more than 20 years. Regarding academic titles, 32.5% of participants were residents, 31.1% were specialists, 11.9% were assistant professors, 10.6% were associate professors, and 13.9% were professors.

According to the participants’ self-assessment of their knowledge level regarding AI, 39.1% rated their knowledge as average, 16.6% as above average, 10.6% as good, and 3.3% as excellent. Meanwhile, 26.8% reported having a little knowledge, and 3.6% stated that they had no idea. When participants were grouped by age under 35 years (*n* = 129), 36–50 years (*n* = 122), and over 50 years (*n* = 51), there was no statistically significant difference in responses among the groups (*p* = 0.232). In the first group, 53 participants (41.1%) described their knowledge as average and 16 (12.4%) as above average; in the second group, 42 (34.4%) reported average and 24 (19.7%) above average knowledge; and in the third group, 23 (45.1%) described their knowledge as average and 10 (19.6%) as above average. However, when participants were categorized according to academic title—those with academic positions (assistant lecturer, associate professors, and professors) versus those without (medical specialization student, specialist physician)—there was a statistically significant increase in the proportion of participants with academic titles who rated their knowledge as above average (*p* = 0.002; Odds Ratio: 3.23; 95% CI: 1.73–6.02).

When asked about the use of AI applications in clinical practice, 58.6% of participants responded as they had never used AI. Participants with academic titles were significantly more likely to report using AI applications in clinical practice than those without academic titles (*p* < 0.001; OR: 2.63; 95% CI: 1.64–4.35). To further investigate factors associated with physicians’ perceptions of artificial intelligence, additional subgroup analyses were performed according to specialty, academic title, age group, professional experience, and previous AI use (Table 2). Specialty was significantly associated with self-perceived AI knowledge, perceived clinical benefits of AI, its expected contribution to healthcare delivery, and overall attitudes toward AI (all *p* < 0.05). Academic title and professional experience were significantly associated only with self-perceived AI knowledge, whereas no significant differences were observed across age groups for the main survey outcomes. Previous experience with AI applications was significantly associated with all major survey outcomes evaluated (all *p* < 0.001).

**Table 2:** Subgroup analyses of clinically relevant survey items according to participant characteristics

Survey item	Specialty *p*	Academic title *p*	Age group *p*	Professional experience *p*	Previous AI use *p*
Self-perceived knowledge about AI	**0.034**	**0.002**	0.232	**0.041**	**< 0.001**
AI will be actively used in the future	0.702	0.113	0.930	0.789	**< 0.001**
AI applications will be beneficial	**0.048**	0.347	0.061	0.786	**< 0.001**
AI will improve healthcare delivery	**0.022**	0.584	0.138	0.531	**< 0.001**
Physicians can easily adapt to AI	0.316	0.054	0.096	0.073	**< 0.001**
Overall opinion on AI applications	**0.016**	0.761	0.095	0.529	**< 0.001**
Overall opinion on AI-assisted medicine	0.110	0.319	0.369	0.282	**< 0.001**

To identify factors independently associated with a positive overall attitude toward artificial intelligence, a multivariable binary logistic regression analysis was performed including age group, sex, specialty, academic title, professional experience, and previous AI use. Previous AI use was independently associated with a positive overall attitude toward AI (adjusted OR for no previous AI use vs. previous AI use: 0.03, 95% CI: 0.004–0.21, *p* < 0.001). Specialty was also an independent predictor, with thoracic surgeons demonstrating higher odds of a positive attitude than pulmonologists (adjusted OR: 3.97, 95% CI: 1.47–10.74, *p* = 0.007). Increasing age group was associated with lower odds of a positive attitude toward AI (adjusted OR: 0.35, 95% CI: 0.15–0.79, *p* = 0.011). Sex, academic title, and professional experience were not independently associated with positive attitudes toward AI (Table 3).

**Table 3:** Multivariate Analysis

Variable	Adjusted OR	95% CI	*p*
Age group	**0.35**	0.15–0.79	**0.011**
Sex (male vs. female)	0.89	0.41–1.96	0.781
Specialty (thoracic surgery vs. pulmonology)	**3.97**	1.47–10.74	**0.007**
Academic title	1.42	0.53–3.77	0.487
Professional experience	1.22	0.82–1.83	0.331
Previous AI use (No vs. Yes)*	**0.03**	0.004–0.21	**< 0.001**

**Fig. 1:**
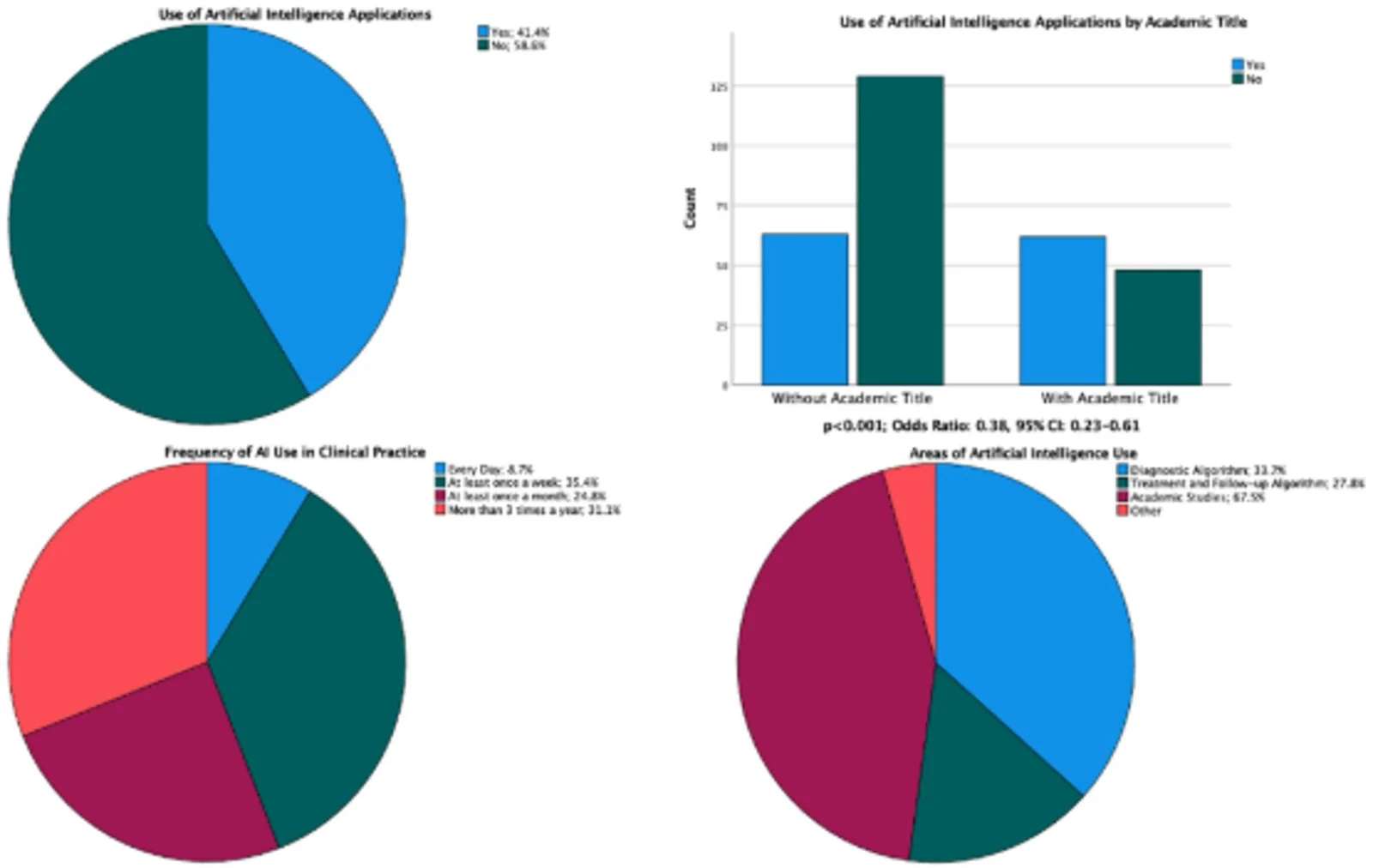
Distribution of participants by AI usage rate and areas of application

Among the 125 participants who reported using AI applications, 35.4% stated that they used them at least once a week, 24.8% at least once a month, 31.1% more than three times a year, and 8.7% every day. The most common areas of AI use were academic research (67.5%), diagnostic algorithms (33.7%), and treatment or monitoring algorithms (27.8%) (Fig. 1).

**Fig. 2:**
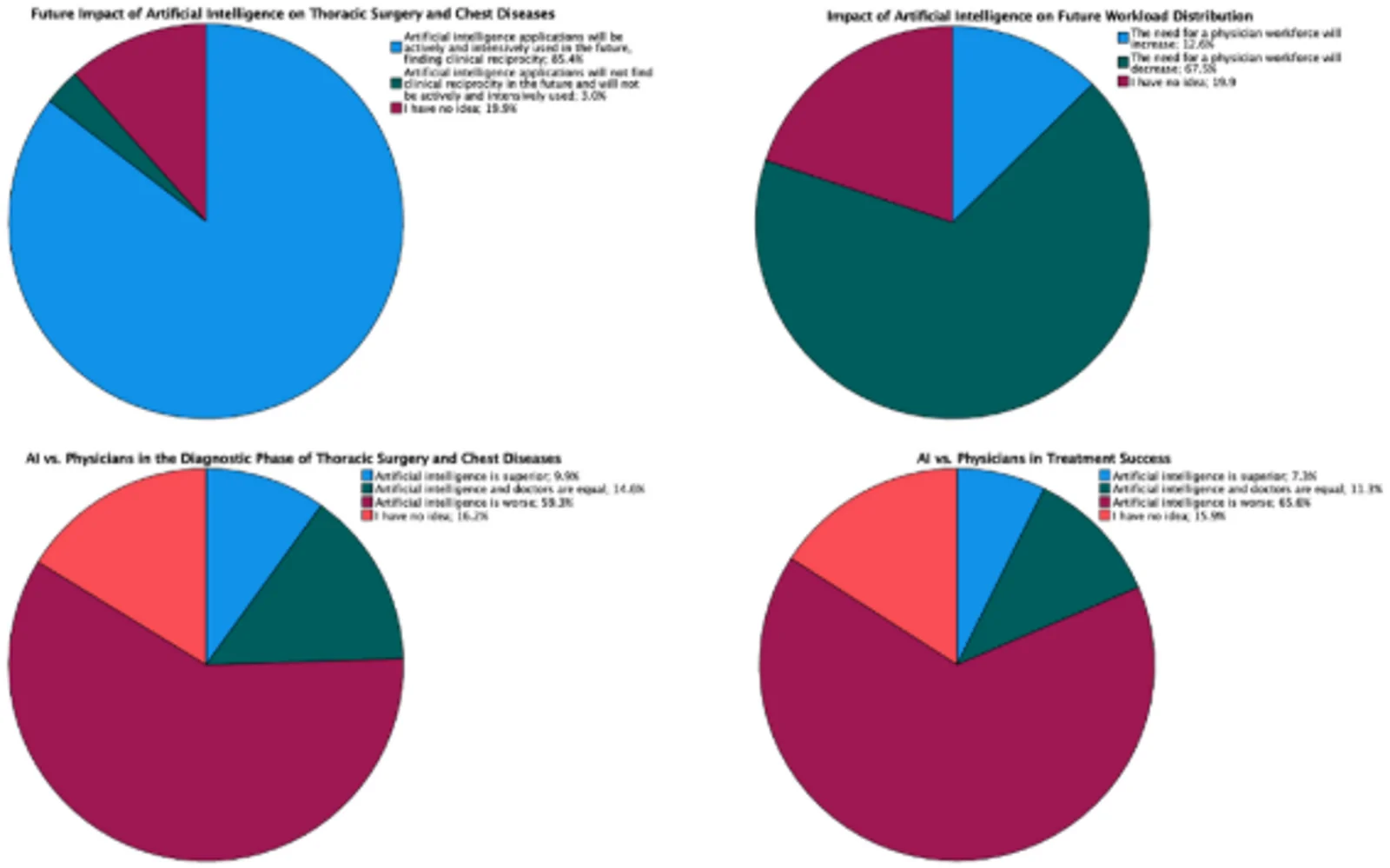
Participants’ evaluations of AI’s future use, impact on workforce, and effectiveness in diagnosis and treatment

A total of 85.4% of participants responded that they believe AI applications will be actively used in clinical practice in the future. Additionally, 67.5% believed that the use of AI would reduce the need for physician workforce. The areas where AI is expected to be most effective in the fields of pulmonology and thoracic surgery include radiology (94.7%), risk analysis (65.2%), evaluation of pulmonary function tests (62.6%), treatment and prognosis estimation (57.9%), diagnosis and treatment of sleep disorders (45.7%), and robotic surgery (42.7%). Furthermore, 59.3% of participants believed that AI applications were less effective than physicians in diagnosis, and 65.6% believed they were less effective in treatment decision-making (Fig. 2).

**Fig. 3:**
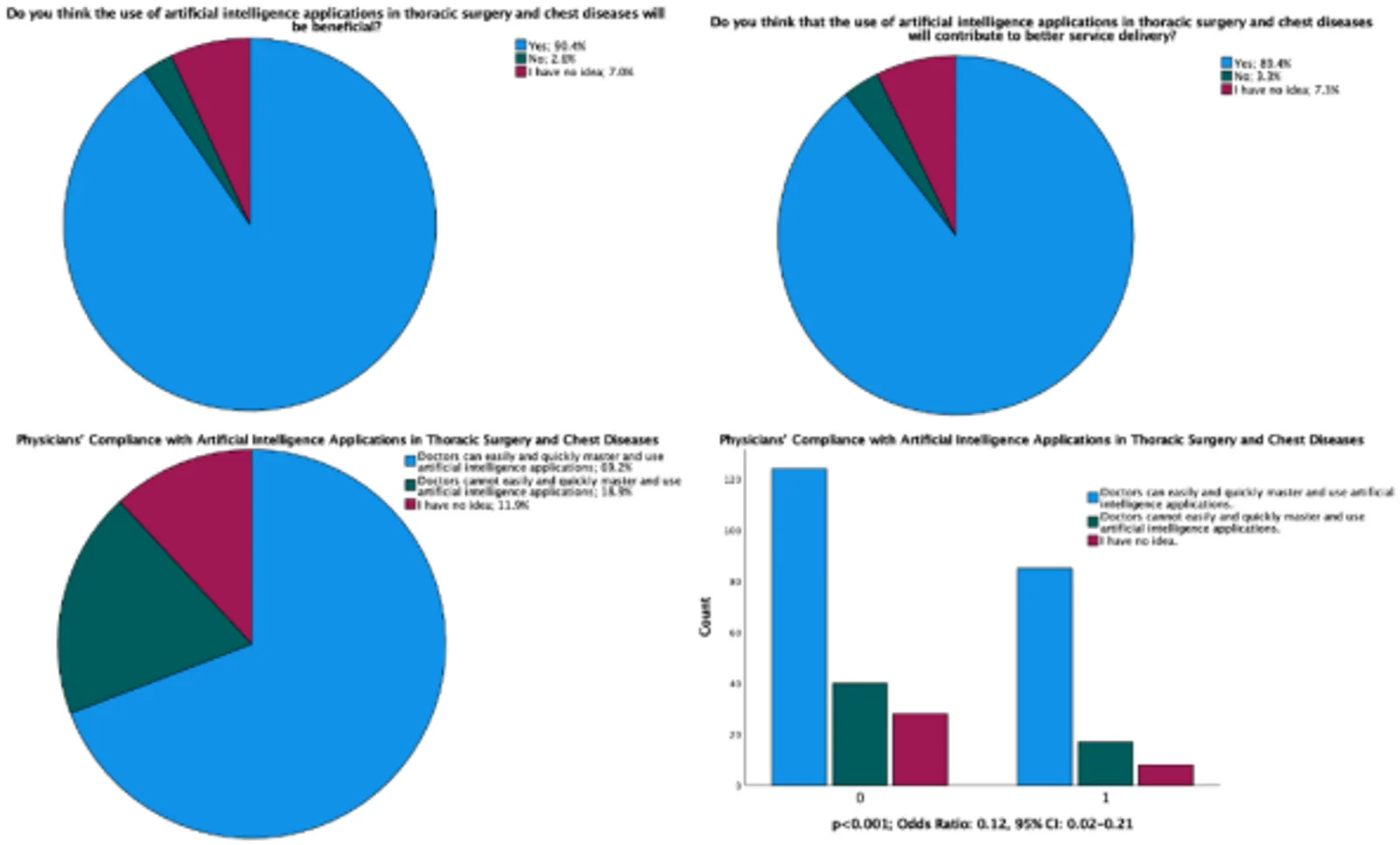
Participants’ evaluations of AI’s benefits, contribution to healthcare delivery, and physicians’ adaptation to AI use

However, 90.4% of participants stated that AI applications would be beneficial, 89.4% believed that they would contribute to healthcare delivery, and 69.2% stated that physicians could adapt to AI quickly and easily. Participants without academic roles were statistically significantly more likely to state that physicians would not be able to adapt to AI quickly and easily (*p* < 0.001; Odds Ratio: 0.12; 95% CI: 0.02–0.21) (Fig. 3).

**Fig. 4:**
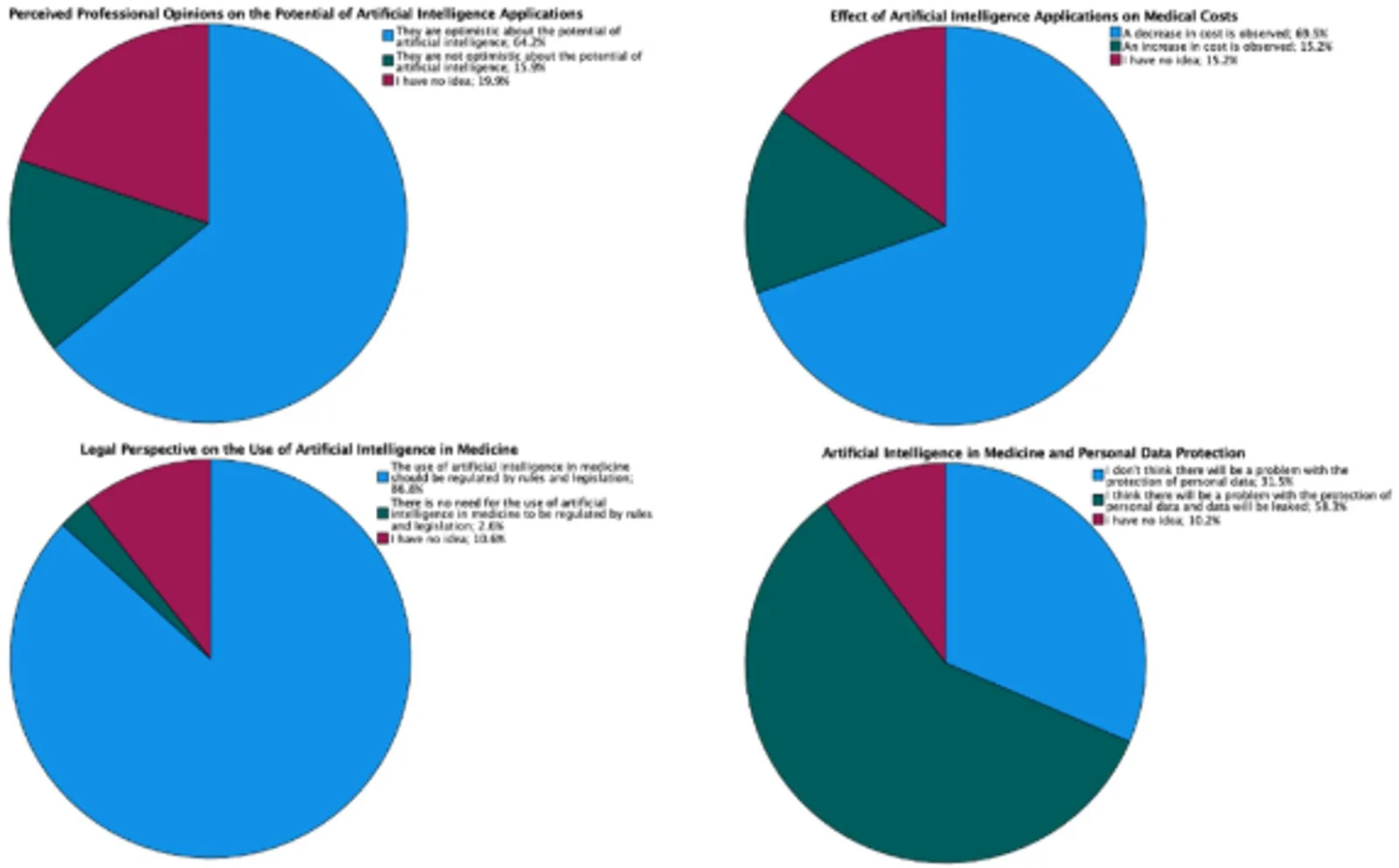
Participants’ evaluations of AI’s potential, cost impact, legal aspects, and data privacy concerns

**Fig. 5:**
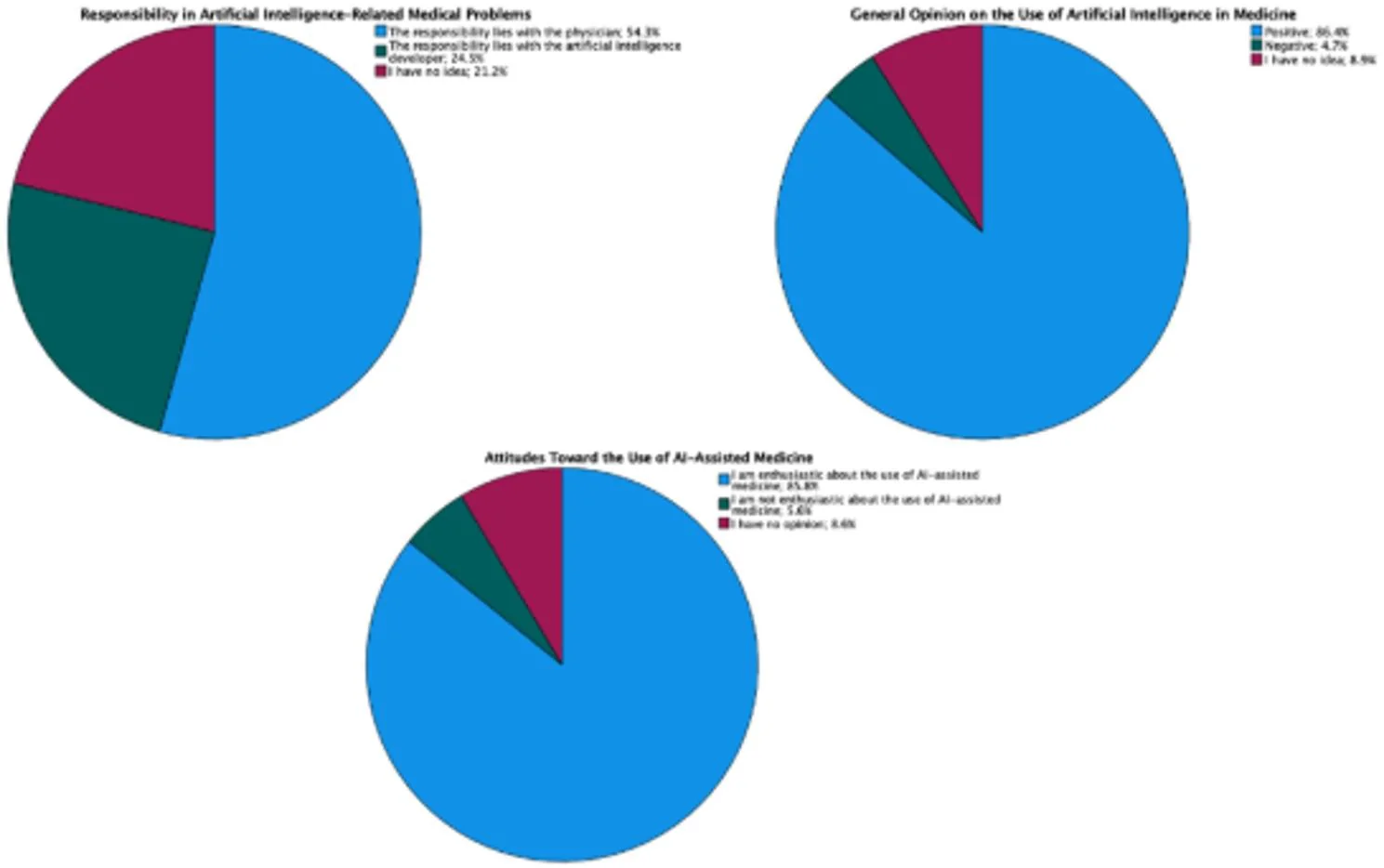
Participants’ evaluations of responsibility for problems related to AI use and the use of AI in medicine

Of all participants, 64.2% stated that physicians are optimistic about the potential of AI. Furthermore, 69.5% believed that the use of AI would reduce costs. A total of 86.8% argued that the use of AI should be governed by legislation and regulations, while 58.3% expressed concerns regarding personal data privacy (Fig. 4). In addition, 54.3% of participants believed that physicians would be held accountable for potential errors. Overall, 86.4% of participants expressed a positive outlook on the use of AI in medicine, and 85.8% reported feeling enthusiastic about it (Fig. 5).

## Discussion

The growing interest in and development of AI applications in healthcare bring not only substantial opportunities but also important ethical, legal, and practical challenges [17, 21, 22]. Although several previous surveys have evaluated the knowledge and perceptions of AI among medical students and healthcare professionals, our study specifically focuses on pulmonologists and thoracic surgeons. Given the increasing role of AI in thoracic imaging, bronchoscopy, robotic surgery, and clinical decision-making, this specialty-specific approach provides valuable insights into the current level of awareness, attitudes, and expectations regarding AI among physicians working in thoracic medicine. These findings contribute to the existing literature by providing specialty-specific data that may help guide future educational initiatives and the responsible implementation of AI in thoracic medicine.

The questionnaire used in this study was not designed to objectively assess participants’ knowledge of artificial intelligence or their clinical competence in using AI. Instead, it evaluated physicians’ self-perceived knowledge, awareness, attitudes, expectations, and experiences regarding AI applications. Consequently, the findings are based on self-reported assessments and may not necessarily reflect participants’ actual knowledge or clinical competence.

Chauhan and colleagues emphasized that the use of AI in diagnostic algorithms and treatment processes could reduce demand for physician workforce and accelerate clinical decision-making [17–19]. In our study, 67.5% of participants believed that the use of AI would reduce physician workforce requirements. This finding suggests that AI could not only improve the efficiency of healthcare systems but also contribute to the delivery of better patient care.

Etienne and colleagues emphasized that the use of AI in clinical practice will continue to increase in the future [20]. Similarly, in our study, the vast majority of participants (85.4%) believed that AI applications will be actively used in clinical practice in the coming years. Furthermore, the statistically significantly higher rate of participants with academic functions who believed that AI will be used in clinical practice demonstrates the growing impact and acceptance of AI in medicine. These findings highlight the potential of AI to revolutionize healthcare delivery.

However, 59.3% of participants believed that AI applications are less effective than physicians in making diagnoses, and 65.6% believed they are less effective than physicians in making treatment decisions. This reflects still existing concerns for AI, replacing human work potential. Doubts regarding whether AI systems are reliable enough to evaluate complex and variable clinical scenarios may explain physicians’ cautious approach toward these technologies. Furthermore, inaccuracies that may occur in cases of incomplete data undermine physicians’ confidence in the potential of AI [21, 22].

The multivariable analysis demonstrated that previous AI use was independently associated with a more positive attitude toward artificial intelligence, even after adjustment for demographic and professional characteristics. This finding suggests that familiarity with AI technologies may facilitate physicians’ acceptance of AI in clinical practice. In addition, thoracic surgeons were more likely than pulmonologists to report positive attitudes toward AI, while increasing age was independently associated with less favorable attitudes. These findings indicate that physicians’ perceptions of AI are influenced by both professional background and prior experience with AI technologies.

Hogan and colleagues emphasized the importance of addressing the ethical and legal dimensions of AI applications, noting that various ethical issues could arise in clinical practice [23, 24]. In our study, 86.8% of participants advocated that the use of AI should be regulated through specific legislation and guidelines, while 58.3% expressed concerns about personal data privacy. The protection of health data is a key factor influencing the reliability and acceptance of AI systems. Moreover, uncertainty surrounding physicians’ accountability in AI-assisted decision-making processes and the legal framework that would apply in cases of potential errors complicates the adoption of this technology.

Bisdas and colleagues argued that integrating AI into medical education would help physicians develop the necessary awareness and skills in this area [22]. The general consensus among participants that AI should be incorporated into medical education could enhance future physicians’ ability to adapt to these technologies. In our study, the statistically significantly higher proportion of academic participants who believed that physicians would adapt more easily to AI supports this finding. Physicians’ overall positive attitudes toward AI applications underscore the importance of incorporating AI into undergraduate, postgraduate, and continuing medical education curricula.

The generally positive attitudes observed in our study suggest that both pulmonologists and thoracic surgeons recognize the growing importance of artificial intelligence in clinical practice. However, despite these positive perceptions, many participants reported only moderate self-perceived knowledge of AI, highlighting an important educational gap. These findings suggest that future educational initiatives should focus not only on general AI concepts but also on specialty-specific applications, including thoracic imaging, pulmonary nodule detection and management, lung cancer diagnosis, pulmonary function test interpretation, perioperative risk prediction, and robotic-assisted thoracic surgery. As AI becomes increasingly integrated into these areas of thoracic medicine, physicians will also require education regarding ethical principles, data privacy, transparency, and medico-legal responsibilities related to AI-assisted clinical decision-making. Such targeted educational strategies may facilitate the safe, effective, and responsible integration of AI technologies into routine pulmonary medicine and thoracic surgery practice.

This study has several limitations. Because data were collected through a voluntary online survey, response and selection biases cannot be excluded. In addition, participants were recruited using a convenience sampling strategy, and the survey link was distributed through multiple electronic communication channels. Because the total number of invited physicians was not recorded, the response rate could not be calculated. This limitation makes it difficult to assess the representativeness of the study sample and increases the potential for selection bias. Physicians with a greater interest in research or digital technologies may also have been more likely to participate, potentially resulting in an overrepresentation of academically active respondents and more favorable perceptions of AI. Furthermore, the questionnaire assessed physicians’ self-perceived knowledge, awareness, and attitudes rather than their objective knowledge or clinical competence regarding AI. Because approximately two-thirds of the participants were pulmonologists, the findings related to thoracic surgeons should be interpreted with caution and should not be considered representative of the entire thoracic surgery community. Finally, because this study was conducted within a single national healthcare setting, the findings may not be fully generalizable to other healthcare systems with different educational environments, regulatory frameworks, or levels of AI adoption.

Furthermore, the questionnaire assessed self-perceived knowledge and attitudes rather than objective competence. Future multicenter or longitudinal studies could provide deeper insights into the evolving role of AI in thoracic medicine. In addition, the rapidly evolving nature of artificial intelligence should be considered when interpreting our findings. At present, AI applications have not yet been uniformly integrated into routine clinical practice, and structured education, institutional implementation, and guidance from professional societies remain limited in many settings. Therefore, the attitudes and awareness reported in this survey likely reflect an early stage of AI adoption. Repeating similar surveys as AI becomes more widely implemented and as physicians gain greater practical experience with these technologies may provide valuable insights into how perceptions, acceptance, and patterns of AI use evolve over time. Participants were recruited using a convenience sampling strategy, and the survey link was distributed through multiple electronic communication channels. Because the total number of invited physicians was not recorded, the response rate could not be calculated. This limitation makes it difficult to assess the representativeness of the sample and increases the potential for selection bias.

In conclusion, this national survey demonstrated that pulmonologists and thoracic surgeons generally have a positive yet cautious attitude toward the use of artificial intelligence in clinical practice. Although most participants anticipated an increasing role for AI in the future, they also expressed important concerns regarding ethical issues, legal responsibility, and data privacy. These findings reflect the current perceptions of the surveyed physicians rather than objective predictions regarding the future implementation of AI. Understanding these perceptions may help guide future educational initiatives, inform policy development, and support the responsible integration of AI into pulmonary medicine and thoracic surgery.

## Supplementary Information

Supplementary Material 1

## Data Availability

No datasets were generated or analysed during the current study.
