# Supplementary material for: Awareness and perceptions of artificial intelligence among pulmonologists and thoracic surgeons: a national survey
Source: J Cardiothorac Surg. 2026 Aug 25;21:701. doi: 10.1186/s13019-026-04672-y (PMC13576169; doi:10.1186/s13019-026-04672-y)
Supplement: Supplementary file 1 [file 13019_2026_4672_MOESM1_ESM.docx]

**Supplementary Table 1:** Survey questions and participants’ responses

| **Questions** | **n (%)** |
| --- | --- |
| **Q 8a:**If you have used artificial intelligence applications in your clinical practice, can you please specify how often?   A) Every day B) At least once a week C) At least once a month D) More than 3 times a year | 14 (8.7%)  **57 (35.4%)**  40 (24.8%)  50 (31.1%) |
| **Q 8b:** Could you please specify in which area you use artificial intelligence applications in your clinical practice?   A) Diagnostic Algorithm B) Treatment and Follow-up Algorithm C) Academic studies D) Other (please specify) | 57 (33.7%)  47 (27.8%)  **114 (67.5%)**  13 (7.7%) |
| **Q 9:** How will artificial intelligence applications affect thoracic surgery and chest diseases in the future?   A) Artificial intelligence applications will be actively and intensively used in the future, finding clinical reciprocity. B) Artificial intelligence applications will not find clinical reciprocity in the future and will not be actively and intensively used. C) I have no idea. | **258 (85.4%)**  9 (3.0%)  35 (11.6%) |
| **Q 10:**How do you expect the workload distribution in thoracic surgery and thoracic diseases to be in the future with the impact of artificial intelligence applications?   A) The need for a physician workforce will increase. B) The need for a physician workforce will decrease. C) I have no idea. | 38 (12.6%)  **204 (67.5%)**  60 (19.9%) |
| **Q 11:** Which field do you think will be most effective in thoracic surgery and thoracic diseases with artificial intelligence applications? (more than one selection is possible)   A) Radiology, Image analysis B) Reconstruction C) Treatment selection D) Risk analysis E) Treatment success -Prognosis prediction F) Robotic surgery G) Respiratory function test analysis H) Intensive care procedures I) Diagnosis and treatment of sleep disorders J) Asthma COPD treatment K) IPF disease | **286 (94.7%)**  77 (25.5%)  118 (39.1%)  197 (65.2%)  175 (57.9%)  129 (42.7%)  189 (62.6%)  56 (18.5%)  138 (45.7%)  57 (18.9%)  74 (24.5%) |
| **Q 12:** In what position do you evaluate artificial intelligence applications when compared to doctors in the **diagnosis** phase of thoracic surgery and chest diseases?   A) Artificial intelligence is superior. B) Artificial intelligence and doctors are equal. C) Artificial intelligence is worse. D) I have no idea. | 30 (9.9%)  44 (14.6%)  **179 (59.3%)**  49 (16.2%) |
| **Q 13:** In what position do you evaluate artificial intelligence applications in thoracic surgery and thoracic diseases when compared to doctors in **treatment** decisions?   A) Artificial intelligence is superior. B) Artificial intelligence and doctors are equal. C) Artificial intelligence is worse. D) I have no idea. | 22 (7.3%)  34 (11.3%)  **198 (65.6%)**  48 (15.9%) |
| **Q 14:**Do you think the use of artificial intelligence applications in thoracic surgery and chest diseases will be beneficial?   A) Yes B) No C) I have no idea. | **273 (90.4%)**  8 (2.6%)  21 (7.0%) |
| **Q 15:** Do you think that the use of artificial intelligence applications in thoracic surgery and chest diseases will contribute to better service delivery?   A) Yes B) No C) I have no idea. | **270 (89.4%)**  10 (3.3%)  22 (7.3%) |
| **Q 16:** What do you think about doctors' compliance with artificial intelligence applications integrated into thoracic surgery and chest diseases clinics?   A) Doctors **can** easily and quickly master and use artificial intelligence applications. B) Doctors **cannot** easily and quickly master and use artificial intelligence applications. C) I have no idea. | **209 (69.2%)**  57 (18.9%)  36 (11.9%) |
| **Q 17:** What do you think thoracic surgeons and chest diseases physicians (independent of your own opinion) think about the potential of artificial intelligence applications?   A) They are **optimistic** about the potential of artificial intelligence. B) They are **not optimistic** about the potential of artificial intelligence. C) I have no idea. | **194 (64.2%)**  48 (15.9%9  60 (19.9%) |
| **Q 18:** How do you evaluate the cost relationship with the increasing use of artificial intelligence applications in medicine?   A) A **decrease** in cost is observed. B) An **increase** in cost is observed. C) I have no idea. | **210 (69.5%)**  46 (15.2%)  46 (15.2%) |
| **Q 19:** What do you think about the legal aspect of the use of artificial intelligence applications in medicine?   A) The use of artificial intelligence in medicine should be regulated by rules and legislation. B) There is no need for the use of artificial intelligence in medicine to be regulated by rules and legislation. C) I have no idea. | **262 (86.8%)**  8 (2.6%)  32 (10.6%) |
| **Q 20:** What do you think about the use of artificial intelligence applications in medicine and the protection of personal data?   A) I don't think there will be a problem with the protection of personal data. B) I think there will be a problem with the protection of personal data and data will be leaked. C) I have no idea. | 95 (31.5%)  **176 (58.3%)**  31 (10.2%) |
| **Q 21:** What do you think about responsibility for problems that will arise with the use of artificial intelligence applications in medicine?   A) The responsibility lies with the **physician.** B) The responsibility lies with the **artificial intelligence developer.** C) I have no idea. | **164 (54.3%)**  74 (24.5%)  64 (21.2%) |
| **Q 22:** What is your general opinion on the use of AI applications in medicine?   A) Positive B) Negative C) I have no idea. | **261 (86.4%)**  14 (4.7%)  27 (8.9%) |
| **Q 23:** What is your general opinion on the use of AI-assisted medicine?   A) I am enthusiastic about the use of AI-assisted medicine. B) I am not enthusiastic about the use of AI-assisted medicine. C) I have no opinion. | **259 (85.8%)**  17 (5.6%)  26 (8.6%) |
